# Contribution of MMP14-expressing cancer-associated fibroblasts in the tumor immune microenvironment to progression of colorectal cancer

**DOI:** 10.3389/fonc.2022.956270

**Published:** 2022-08-16

**Authors:** Yusuke Makutani, Hisato Kawakami, Takahiro Tsujikawa, Kanako Yoshimura, Yasutaka Chiba, Akihiko Ito, Junichiro Kawamura, Koji Haratani, Kazuhiko Nakagawa

**Affiliations:** ^1^ Department of Surgery, Kindai University Faculty of Medicine, Osaka-Sayama, Japan; ^2^ Department of Medical Oncology, Kindai University Faculty of Medicine, Osaka-Sayama, Japan; ^3^ Department of Otolaryngology–Head and Neck Surgery, Kyoto Prefectural University of Medicine, Kyoto, Japan; ^4^ Clinical Research Center, Kindai University Hospital, Osaka-Sayama, Japan; ^5^ Department of Pathology, Kindai University Faculty of Medicine, Osaka-Sayama, Japan

**Keywords:** colorectal cancer, matrix metalloproteinase 14 (MMP14), multiplex immunohistochemistry (mIHC), cancer-associated fibroblast (CAF), M2 tumor-associated macrophages (M2-TAMs)

## Abstract

Matrix metalloproteinase 14 (MMP14) expression is implicated in progression of colorectal cancer, but its role in the tumor microenvironment (TME) has been unclear. The relevance of MMP14 to colorectal cancer progression was explored by analysis of transcriptomic data for colorectal adenocarcinoma patients (*n* = 592) in The Cancer Genome Atlas. The role of MMP14 in the TME was investigated in a retrospective analysis of tumor samples from 86 individuals with stage III colorectal cancer by single cell–based spatial profiling of MMP14 expression as performed by 12-color multiplex immunohistochemistry (mIHC). Analysis of gene expression data revealed that high *MMP14* expression was associated with tumor progression and implicated both cancer-associated fibroblasts (CAFs) and tumor-associated macrophages in such progression. Spatial profiling by mIHC revealed that a higher percentage of MMP14^+^ cells among intratumoral CAFs (MMP14^+^ CAF/CAF ratio) was associated with poorer relapse-free survival. Multivariable analysis including key clinical factors identified the MMP14^+^ CAF/CAF ratio as an independent poor prognostic factor. Moreover, the patient subset with both a high MMP14^+^ CAF/CAF ratio and a low tumor-infiltrating lymphocyte density showed the worst prognosis. Our results suggest that MMP14^+^ CAFs play an important role in progression of stage III colorectal cancer and may therefore be a promising therapeutic target.

## Introduction

Colorectal cancer is the second most common cause of cancer-related deaths worldwide (1). Stage III colorectal cancer, which accounts for one-third of all colorectal cancer cases at diagnosis, is generally characterized by local peritoneal invasion or lymph node metastasis and thus has a high recurrence rate of >30% even after complete tumor resection (2, 3). Although 5-fluorouracil (5-FU)–based adjuvant chemotherapy is administered to mitigate this poor prognosis in individuals with stage III colorectal cancer, no substantial advances in treatment have been achieved in recent decades and treatment outcome remains unsatisfactory (4–6). Optimization of treatment strategies for stage III colorectal cancer, including better stratification of patients, is therefore an important clinical goal.

Increasing evidence has suggested that the tumor microenvironment (TME), including immune cells and cancer-associated fibroblasts (CAFs), plays an important role in cancer fate including therapeutic response and clinical outcome (7–14). An Immunoscore based on peritumoral and intratumoral populations of T cells including CD3^+^ and CD8^+^ T cells has been found to classify stage III colorectal cancer into recurrent and nonrecurrent subgroups (15–19). In addition, transcriptome-based phenotyping allows categorization of colorectal cancer into four distinct subgroups, designated Consensus Molecular Subtypes (CMSs). Similar to the Immunoscore, the CMS classification has revealed that a gene expression profile suggestive of intratumoral immune infiltration (CMS1) predicts a better relapse-free survival (RFS), whereas poorly immunogenic profiles including a canonical subtype characterized by WNT and MYC pathway activation (CMS2) and a metabolic subtype characterized by metabolic dysregulation (CMS3) tend to be associated with recurrence after complete tumor resection. A mesenchymal phenotype characterized by enrichment of transcriptomes associated with stromal components and angiogenesis (CMS4) shows the worst RFS, even though the tumors manifest moderate immune-related gene expression (20). CAFs constitute one such stromal component and are key players in the TME (21). These cells are defined as activated fibroblasts present specifically in the TME (22), and they are thought to interact with immune cells such as tumor-associated macrophages (TAMs) and myeloid-derived suppressor cells (MDSCs) (22–29). These findings suggest that further investigation of the TME, including both immune cells and mesenchymal or other stromal components, is required to better characterize the biology of colorectal cancer and to inform the development of new treatment strategies based on a better stratification of stage III colorectal cancer.

Matrix metalloproteinase 14 (MMP14) is a transmembrane proteolytic enzyme (30) that plays a key role in establishment of a desmoplastic TME in colorectal cancer (31). Indeed, recent clinical studies have shown that colorectal tumors with a relatively high *MMP14* expression level tend to have a mesenchymal phenotype such as CMS4 (20, 32). In addition, a study based on data in The Cancer Genome Atlas (TCGA) found that a high *MMP14* expression level was associated with poor prognosis in stage I–III colorectal cancer (33). Furthermore, a preclinical study of genetically engineered or syngeneic mouse models revealed that the expression of MMP14 in colorectal tumors gave rise to 5-FU resistance through activation of CAFs (32). These various observations suggest that MMP14 expression in tumors may contribute to the recurrence of colorectal cancer in a manner dependent on the TME, and that clarification of this role of MMP14 may lead to improvement in the survival of individuals with stage III colorectal cancer.

We have now conducted a retrospective biomarker analysis of surgically resected tumor specimens from patients with stage III colorectal cancer attending Kindai University Hospital as well as an analysis of TCGA transcriptomic data in order to clarify this role of MMP14. We performed 12-color multiplex immunohistochemistry (mIHC) to evaluate the relation of MMP14 protein expression in multiple cell subsets including intratumoral immune cells to colorectal cancer progression. Our digital pathology platform thus allowed a single cell–based quantitative spatial profiling of MMP14 expression in various cell lineages in the stage III colorectal tumors. We found that CAFs are an important source of MMP14 and that MMP14^+^ CAFs may act in collaboration with TAMs to promote the progression of colorectal cancer. Our results suggest a novel risk stratification of stage III colorectal cancer based on MMP14 expression in CAFs.

## Materials and methods

### Patients

We consecutively reviewed the medical records of patients with stage III colorectal cancer who underwent definitive surgery followed by adjuvant chemotherapy at Kindai University Hospital between January 2013 and December 2017. Patients who received adjuvant chemotherapy for <3 months and those without sufficient tumor tissue available for our study were excluded. Colorectal cancer was staged according to the eighth edition of the Union for International Cancer Control (UICC) TNM Classification of Malignant Tumors (34). From this review, we identified 86 patients who received 5-FU–based adjuvant chemotherapy (**Supplementary Figure S1**). Colorectal cancer for which the primary tumor was located proximal to the splenic flexure was defined as right-sided. RFS was defined as the time from surgery to the date that clinical evidence of recurrent or metastatic disease was obtained or to the date of last follow-up. The cutoff date for follow-up was 13 May 2021.

### mIHC staining protocol

mIHC staining was performed as described previously (35, 36). In brief, sections (thickness, 4 µm) of formalin-fixed, paraffin-embedded (FFPE) tissue were depleted of paraffin, stained with hematoxylin (S3301, Dako), and subjected to whole-tissue scanning with a NanoZoomer instrument (Hamamatsu Photonics) at 20× magnification to detect the nucleus of each cell. Peroxidase activity was blocked by exposure of the sections to 0.6% hydrogen peroxide in phosphate-buffered saline (PBS) for 15 min, and antigen retrieval was performed by exposure to microwave radiation (to achieve a temperature of 95°C for 15 min) in Antigen Retrieval Citra Solution (B-HK0809K, BioGenex). After exposure to 5.0% goat serum and 2.5% bovine serum albumin (37) in PBS to block nonspecific sites, the tissue was incubated with primary antibodies, anti-mouse or anti-rabbit Histofine Simple Stain MAX PO horseradish peroxidase–conjugated polymer (Nichirei Biosciences), and the alcohol-soluble peroxidase substrate 3-aminomethyl carbazolezole as shown in **Supplementary Table S1**. Chromogenic destaining and antibody stripping were performed between sequential staining steps in the order indicated (**Supplementary Table S1**). Representative images of staining for each antigen are shown in **Supplementary Figure S2A**.

### Digital analysis

Representative fields of 1884 by 1884 μm for two distinct tumor areas, the invasive front (IF) and center of the tumor (CT), were randomly selected from the whole digital slides with the use of Aperio ImageScope v.12 software (Leica Biosystems). The IF was defined as the area within 300 µm external to the boundary separating the tumor cell area from the surrounding connective tissue (**Supplementary Figure S2B**), as described previously (38). The CT was defined as an intratumoral area and was further categorized into a tumor cell nest (TN) area and a non–tumor cell area comprising mostly intratumoral stromal tissue (ISA) (**Supplementary Figure S2B**) with the use of a mathematical morphological approach performed with the Tissue Segmentation platform introduced in our previous study (39). Between two and five fields of view were evaluated depending on the size of the tumor (average of 3.64 fields of view for CT and 4.37 for IF). The CT field could not be determined because of the small size of the tumor in one case. Image processing and subsequent computational analysis were performed with ImageJ/Fiji version 1.51 (National Institutes of Health), CellProfiler version 2.2.0 (Broad Institute), Aperio ImageScope, and FCS Express 7 Image Cytometry v.7.04.0014 (*De Novo* Software) as described previously (35, 36). In brief, the serially scanned images were coregistered by ImageJ/Fiji and CellProfiler. The coregistered images were then converted to single-marker images by ImageJ. Pseudocoloring of the images was performed in Aperio ImageScope. Quantitative assessment of the images was performed with FCS Express 7 Image Cytometry after single-cell segmentation and quantification of staining intensity by CellProfiler. For quantification of individual immune cell subsets by FCS Express 7 Image Cytometry, fluorescence-minus-one controls were used to determine true positive cells. The final value for each cell number was calculated as the average of values from the multiple fields of view.

### Immunoscore

The Immunoscore was determined as described previously (16). In brief, the densities of CD3^+^ and CD8^+^ T cells in both the CT or ISA and the IF regions were calculated, and tumors were then categorized into two groups based on the median values of the densities of each T cell subset in each region. Values lower than or equal to the median and those higher than the median were scored as 0 or 1, respectively. The sum of all scores (CD3^+^ cells in CT or ISA, CD3^+^ cells in IF, CD8^+^ cells in CT or ISA, and CD8^+^ cells in IF) was calculated as the final Immunoscore. A final score based on CT and IF regions was determined as the conventional Immunoscore. In addition, an IS-Immunoscore based on scores in IF and ISA regions was provided by our Tissue Segmentation platform. Patients with final scores of ≥2 or <2 were classified as having a high or low Immunoscore, respectively.

### Transcriptomic data analysis

Normalized gene expression data (RNA Seq v2) for the colorectal adenocarcinoma cohort (*n* = 592) of the TCGA database were downloaded *via* the cBioportal website (http://www.cbioportal.org) in August 2021. The downloaded value (*x*) was converted to *x* + 1 for subsequent differential gene expression analysis. Genes expressed at a low level (those for which >50% of samples showed an expression value below the minimum threshold defined as 1.5) were filtered out. Gene set enrichment analysis (GSEA) (http://www.broadinstitute.org/gsea/index.jsp) was performed with hallmark gene sets v7.4 and GSEA software version 4.0.3 (Broad Institute) (40). The extent of immune cell infiltration was determined as the absolute score of the LM22 signature with the CIBERSORTx tool (41) (https://cibersortx.stanford.edu). Correlation coefficients for gene expression levels were estimated with the cBioportal website platform.

### Statistical analysis

Differences between survival curves constructed by the Kaplan-Meier method were assessed with the log-rank test. The hazard ratio (HR) and its 95% confidence interval (CI) were calculated according to the Cox proportional hazards model. Correlations were examined with the Spearman rank correlation test. The Benjamini-Hochberg method was applied to calculate the false discovery rate (FDR) for multiple testing unless specified otherwise. The Wilcoxon rank-sum test was adopted to compare continuous variables, with the Bonferroni correction being applied to adjust *P* values in multiple tests. Multivariable analysis was performed with the Cox proportional hazards regression model. Age, sex, tumor location, stage, histology, and adjuvant chemotherapy were chosen as covariates because they were identified as clinically important factors in previous studies (42–45). All *P* values were based on a two-sided hypothesis, and all statistical analysis was performed with JMP software version 15.1.0 (SAS Institute) or GraphPad Prism version 9.1.1 (GraphPad Software).

### Study approval

This retrospective study was approved by the Institutional Review Board (R02-070) and the Ethics Committee of Kindai University Faculty of Medicine. The Institutional Review Board waived the need to obtain written consent.

## Results

### Association of MMP14-related fibrotic tissue with immunosuppressive TAM infiltration into tumors suggested by TCGA data

**Figure 1:**
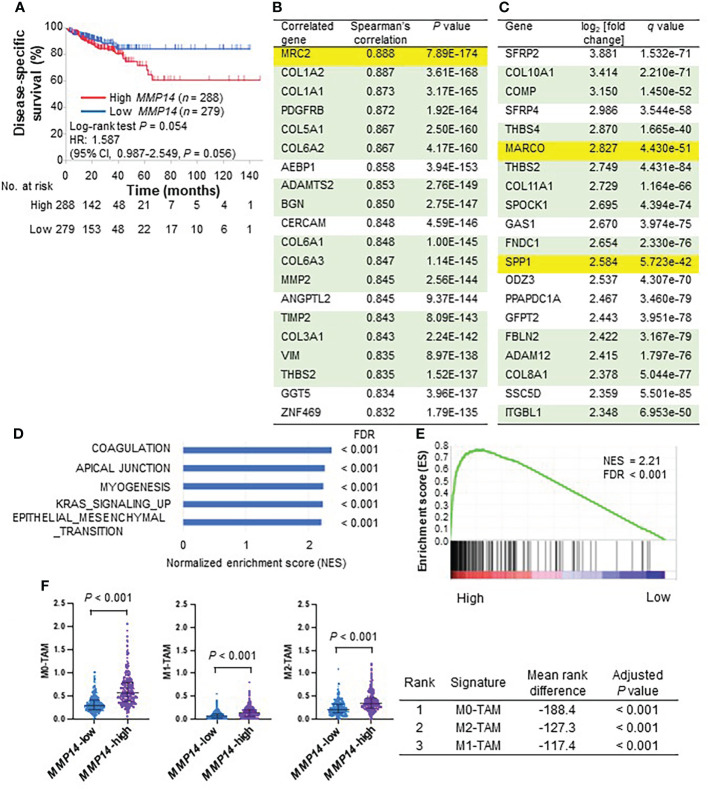
Analysis of the colorectal adenocarcinoma cohort (*n* = 592) of the TCGA database according to *MMP14* expression. **(A)** Kaplan-Meier curves for disease-specific survival in patients divided according to the median value of *MMP14* expression level. Vertical bars denote censoring. **(B)** The top 20 genes whose expression level was correlated with that of *MMP14* ranked according to Spearman’s correlation coefficient. Genes related to stromal tissue or M2-TAMs are highlighted in green and yellow, respectively. **(C)** The top 20 genes whose expression was associated with that of *MMP14* ranked according to log_2_ of the fold change in normalized expression value for *MMP14*-high relative to *MMP14*-low tumors. FDR *q* values were also calculated. Genes are highlighted as in **(B)**. **(D)** The top five hallmark gene sets whose expression was up-regulated in the *MMP14*-high group as revealed by GSEA. FDR *q* values were calculated with GSEA software. **(E)** GSEA plot of enrichment for the gene signature related to epithelial-mesenchymal transition for *MMP14*-high versus *MMP14*-low tumors. **(F)** The top three immune cell signatures associated with *MMP14* expression by CIBERSORTx analysis. In the dot plots for the immune cell signatures (top), each dot represents one patient and the median value and interquartile range are indicated. The mean rank difference values and adjusted *P* values calculated by multiple Wilcoxon rank-sum tests with Bonferroni correction are also shown (bottom).

To explore the clinical relevance of MMP14 in colorectal cancer, we first analyzed publicly available gene expression data for tumor tissue of the colorectal adenocarcinoma data set in TCGA. A high level of *MMP14* expression in tumors was associated with a poor survival outcome in this cohort (**Figure 1A**). To investigate the character of tumors expressing *MMP14* at a high level, we searched for genes whose expression level was correlated with that of *MMP14* (**Figure 1B**). This analysis showed that *MMP14* expression was strongly correlated with that of genes related to stromal cells (including *PDGFRB* and *VIM*) as well as that of many collagen genes (46), suggesting that CAFs might contribute to tumor aggressiveness associated with MMP14. Of note, however, the expression level of *MRC2*, an M2-type immunosuppressive and tumorigenic macrophage–related gene (47), was most strongly correlated with that of *MMP14*, also implicating M2-TAMs in MMP14-related tumor progression. In addition, we generated a list of differentially expressed genes for tumors with high versus low levels of *MMP14* expression by calculating log_2_ of the fold change in expression level and the FDR (*q*) value. This list included M2-TAM–related genes such as *MARCO* (48) and *SPP1* (49, 50) in the top 20 genes associated with high *MMP14* expression (**Figure 1C**). *MRC2* was also a gene associated with high *MMP14* expression in this list (ranked 143 out of 16,795 genes, log_2_[fold change] = 1.729, *q* value = 3.999 × 10^–87^). *MMP14* expression was also associated with that of other M2-TAM genes such as *CD163* (ranked 103, log_2_[fold change] = 1.815, *q* value = 2.501 × 10^–40^) and *CD206* (ranked 327, log_2_[fold change] = 1.475, *q* value = 4.332 × 10^–27^).

We next performed gene expression profiling of *MMP14*-expressing colorectal tumors with the use of GSEA and CIBERSORTx analysis. GSEA revealed that a signature enriched in fibroblast-related genes (HALLMARK_EPITHELIAL_MESENCHYMAL_TRANSITION) was in the top five gene sets that were significantly up-regulated in tumors expressing *MMP14* at a high level (**Figures 1D, E**). CIBERSORTx analysis of the LM22 signature matrix suggested that *MMP14* expression was associated with the accumulation of TAMs including M2-TAMs in colorectal tumors, with the top three immune cell subsets identified by this analysis being M0-, M2-, and M1-TAMs, respectively (**Figure 1F**). Overall, these results suggested that *MMP14* expression, in association with CAFs and M2-TAM infiltration into tumor tissue, contributes to poor survival outcome in patients with colorectal cancer.

### Contribution of MMP14 expression in CAFs to colorectal cancer recurrence as revealed by spatial profiling with mIHC

To examine further how *MMP14* expression in tumors might contribute to poor survival outcome in colorectal cancer, we performed 12-color mIHC analysis of tumor tissue surgically removed from patients with stage III colorectal cancer at Kindai University Hospital. A consecutive review of medical records from 2013 to 2017 identified 86 individuals who underwent curative resection for stage III colorectal cancer followed by 5-FU–based adjuvant chemotherapy for inclusion in our study (**Table 1**). Most cases were stage IIIB (71%), and all were adenocarcinoma, including 13 cases with a histology known to be prognostic for an unfavorable survival outcome (poorly differentiated, mucinous, and signet ring cell). Postoperative adjuvant chemotherapy regimens included 5-FU administered either alone or together with oxaliplatin in 45 (52%) and 41 (48%) patients, respectively. Twenty-five patients (29%) experienced disease recurrence during follow-up with a median of 60.1 months.

**Table 1:** Clinicopathologic characteristics of the study patients for mIHC (*n* = 86).

Characteristic	No. of patients (%)
[Median age (range), years	67 (31–81)
Sex	
Male	44 (51)
Female	42 (49)
Tumor location	
Right-sided colon	30 (35)
Left-sided colon	20 (23)
Rectum	36 (42)
Stage	
IIIA	10 (12)
IIIB	61 (71)
IIIC	15 (17)
Tumor classification (T)	
T1–2	12 (14)
T3	64 (74)
T4	10 (12)
Lymph node metastasis (N)	
N1	62 (72)
N2	24 (28)
Histology	
Papillary, tubular	73 (85)
Poorly differentiated, mucinous, signet ring cell	13 (15)
Adjuvant chemotherapy	
Oxaliplatin with 5-FU	41 (48)
5-FU	45 (52)
Recurrence	
No	61 (71)
Yes	25 (29)

**Figure 2:**
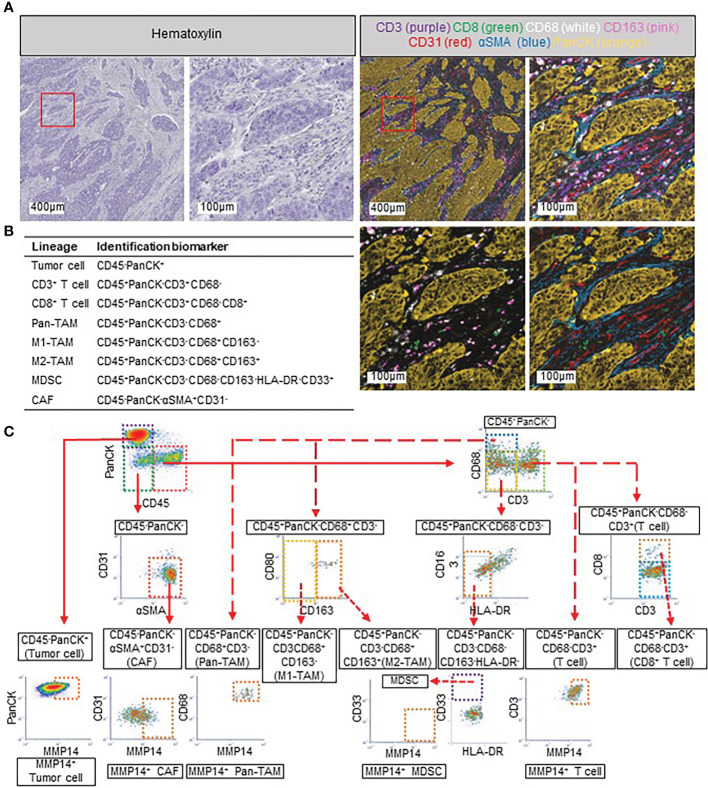
Multiplex immunohistochemistry (mIHC) and strategy for quantitative evaluation of cell populations. **(A)** Representative images of mIHC. Images for hematoxylin staining (upper) and multicolor images (lower) are shown. The area within the red box is shown at higher magnification in the other corresponding images. Seven markers are shown in the top two multicolor images, with CD8 (green), CD68 (white), CD163 (pink), and pan-cytokeratin (panCK, orange) being shown in the lower left image and CD8 (green), CD31 (red), αSMA (blue), and panCK (orange) in the lower right. **(B)** Definition of cell lineages according to marker expression in the present study. **(C)** Gating strategy for mIHC-based single-cell population analysis.

On the basis of our analysis of TCGA gene expression data, we spatially evaluated multiple cell compartments including CAFs, M1- and M2-TAMs, and tumor cells in addition to MMP14 expression in individual FFPE tumor tissue sections by 12-color mIHC. Tumor-infiltrating lymphocytes (TILs) were also evaluated in the same sections, given that previous studies based on the Immunoscore have implicated these cells in colorectal cancer progression (16–19). In addition, we included MDSCs in this analysis because these cells have been associated with TAMs and CAFs in the TME (51–53). Each cell lineage as defined by marker expression was appropriately distinguished by our 12-color mIHC platform (**Figures 2A, B**). Image cytometry–based analysis of cell populations allowed quantitative evaluation of the cell subsets as cell number per area (**Figure 2C**). In addition, we applied our recently developed Tissue Segmentation method (39) to quantify cell number according to different tumor areas including IF, ISA, and TN (**Supplementary Figure S2B**), thus allowing detailed evaluation of the clinical relevance of each cell subset. Given that α smooth muscle actin (αSMA), a marker for CAFs in our mIHC panel, was expressed in both smooth muscle cells and fibroblasts in the IF, this region was excluded from subsequent analysis of the association of CAFs with immune cells. We detected CD45^+^ immune cells in both ISA and TN regions of CT, whereas CAFs and tumor cells were identified almost exclusively in ISA and TN, respectively (**Supplementary Figure S2C**), supporting the ability of our Tissue Segmentation method to distinguish between these two regions of the CT area.

**Figure 3:**
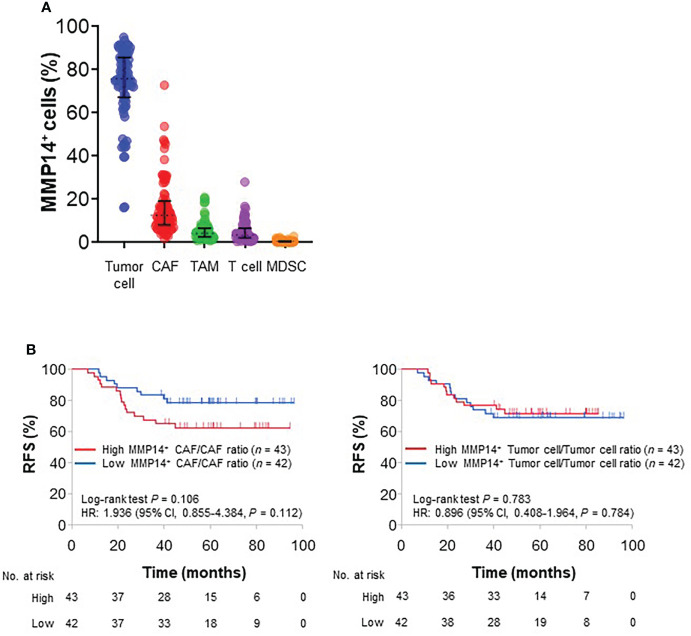
Prognostic impact of MMP14-expressing cells. **(A)** Percentage of MMP14-expressing cells among cell subsets as determined by mIHC analysis. Each dot represents one patient, and the median value and interquartile range are shown for each plot. **(B)** Kaplan-Meier curves for RFS according to the median values of the MMP14^+^ CAF/CAF ratio (left) or the MMP14^+^ tumor cell/tumor cell ratio (right).

To determine the cell lineages responsible for MMP14-related tumor recurrence, we evaluated the relation of RFS to MMP14 expression in different cell types. MMP14 expression was detected predominantly in tumor cells and CAFs, although it was also apparent in TAMs and TILs (**Figure 3A**). A higher percentage of MMP14-expressing cells among CAFs (MMP14^+^ CAF/CAF ratio) was associated with a poorer RFS (HR of 1.936, with a 95% CI of 0.855–4.384), whereas a similar trend was not apparent for MMP14 expression in tumor cells, TAMs, or TILs (**Figure 3B** and **Supplementary Figure S3A**). Overall CAF density was not correlated with the MMP14^+^ CAF/CAF ratio (**Supplementary Figure S3B**) and was not associated with RFS (**Supplementary Figure S3C**). Collectively, our spatial profiling data thus suggested that intratumoral CAFs might contribute to tumor progression in stage III colorectal cancer by expressing MMP14.

### Relation of MMP14 expression in CAFs to the infiltration of M2-TAMs into the TN

**Figure 4:**
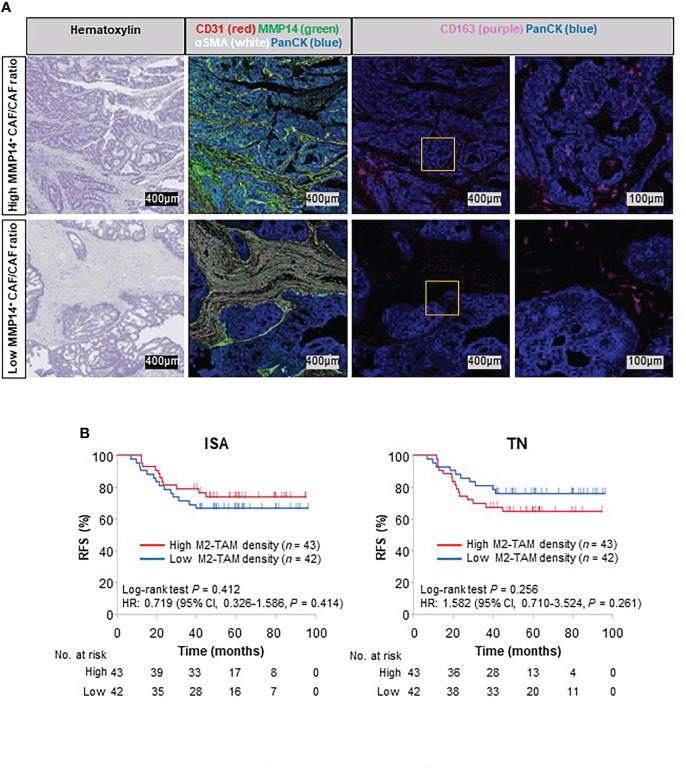
Association of M2-TAM distribution in tumors with MMP14 expression in CAFs and survival outcome. **(A)** Representative mIHC images showing the relation between MMP14-expressing CAFs and the spatial distribution of M2-TAMs. The regions within the yellow squares are shown at higher magnification in the corresponding images to the right. The upper and lower sets of images correspond to patients with a high or low MMP14^+^ CAF/CAF ratio, respectively. **(B)** Kaplan-Meier curves for RFS according to the median values of M2-TAM density in ISA (left) or TN (right) regions.

We next investigated further the relation between TAMs and MMP14^+^ CAFs in stage III colorectal cancer suggested by our analysis of TCGA transcriptomic data. The MMP14^+^ CAF/CAF ratio was positively correlated with M2-TAM density in both the TN and ISA, whereas it was not correlated with the density of M1-TAMs, TILs, or MDSCs (**Supplementary Tables S2, S3**). In contrast, overall CAF density was not positively correlated with M2-TAM density in TN or ISA regions (**Supplementary Figures S3D, E**). These data suggested that M2-TAMs might infiltrate into the TN *via* an intratumoral stromal space enriched in MMP14 expressed by CAFs (**Figure 4A**). We also examined the impact of these tumor-infiltrating M2-TAMs on colorectal cancer recurrence. Whereas the density of M2-TAMs in the ISA was not associated with colorectal cancer recurrence (HR of 0.719, with a 95% CI of 0.326–1.586), a high M2-TAM density in the TN tended to be associated with a poor RFS (HR of 1.582, with a 95% CI of 0.710–3.524) (**Figure 4B**). Together, these results suggested that MMP14-related colorectal cancer aggressiveness might be explained, at least in part, by the promotion of M2-TAM infiltration into the TN by MMP14^+^ CAFs.

### Recurrence of stage III colorectal cancer with a high MMP14^+^ CAF/CAF ratio is exacerbated by a low lymphocyte density in peri- and intratumoral stromal areas

To clarify the clinical importance of MMP14^+^ CAFs in stage III colorectal cancer, we performed a further detailed analysis taking into account both peritumoral and intratumoral lymphocytes, given that the prognostic relevance of these immune cells has been well-validated by the Immunoscore or similar scores in previous studies (15–19). Indeed, a higher conventional Immunoscore was associated with a longer RFS in our study cohort (HR of 0.504, with a 95% CI of 0.210–1.207) (**Supplementary Figure S4A**). However, our Tissue Segmentation method indicated that TILs in the TN were not relevant to tumor recurrence, whereas a higher density of those in stromal areas including the IF and ISA was associated with a longer RFS (**Supplementary Figures S4B, C**), possibly because lymphocytes were distributed mainly in these peritumoral and intratumoral stromal areas rather than in the TN (**Supplementary Figure S4D**). We therefore developed a modified Immunoscore based on CD3^+^ and CD8^+^ cell density only in IF and ISA regions, excluding the TN region that is included in the conventional Immunoscore. This IS-Immunoscore (IF-ISA–Immunoscore) was more clearly prognostic (HR of 0.304, with a 95% CI of 0.134–0.689) (**Supplementary Figure S4E**) than was the conventional Immunoscore (**Supplementary Figure S4A**).

**Figure 5:**
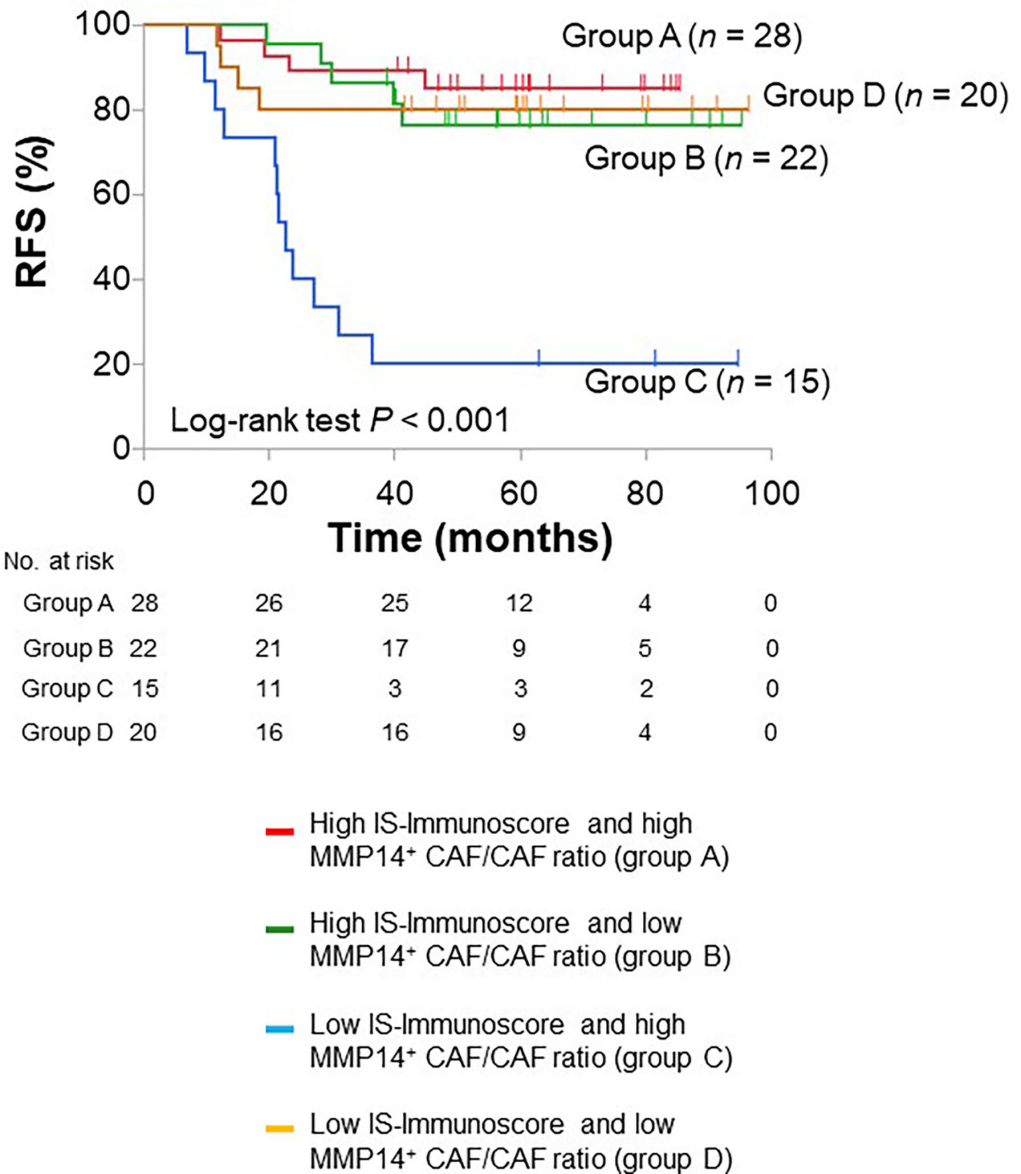
Kaplan-Meier curves for RFS based on the combination of the IS-Immunoscore and MMP14^+^ CAF/CAF ratio. The median value was used as the cutoff for the ratio.

We next performed multivariable analysis including clinically important baseline characteristics as well as the IS-Immunoscore as covariates to test further the prognostic relevance of MMP14^+^ CAFs. This analysis revealed that a high MMP14^+^ CAF/CAF ratio was independently associated with a worse RFS (HR of 2.926, with a 95% CI of 1.167–7.334), whereas a high IS-Immunoscore was an independent prognostic factor for a better RFS (HR of 0.277, with a 95% CI of 0.117–0.652) (**Table 2**). We therefore divided our stage III colorectal cancer patients into four groups according to these two independent biomarkers (IS-Immunoscore and MMP14^+^ CAF/CAF ratio). Among these four groups (high IS-Immunoscore and high MMP14^+^ CAF/CAF ratio, group A; high IS-Immunoscore and low MMP14^+^ CAF/CAF ratio, group B; low IS-Immunoscore and high MMP14^+^ CAF/CAF ratio, group C; low IS-Immunoscore and low MMP14^+^ CAF/CAF ratio, group D), group C showed the worst prognosis whereas the other three groups each showed a similarly better prognosis (**Figure 5**). Comparison of group C with the other three groups combined yielded a HR of 7.382 (95% CI, 3.309–16.469), indicative of the higher prognostic relevance of the combination of the IS-Immunoscore and MMP14^+^ CAF/CAF ratio.

**Table 2:** Univariable and multivariable analysis of clinicopathologic factors for RFS (*n* = 85).

Characteristic	Univariable analysis HR (95% CI)	*P* value	Multivariable analysis HR (95% CI)	*P* value
Age (years)				
<67	1 (reference)	0.267	1 (reference)	0.271
≥67	1.574 (0.706–3.507)		1.612 (0.688–3.774)	
Sex				
Male	1 (reference)	0.968	1 (reference)	0.875
Female	1.016 (0.463–2.227)		0.931 (0.383–2.262)	
Location				
Right-sided colon	1 (reference)	0.449	1 (reference)	0.281
Left-sided colon/rectum	1.401 (0.585–3.355)		1.684 (0.653–4.346)	
Stage				
IIIA/IIIB	1 (reference)	0.240	1 (reference)	0.055
IIIC	1.733		2.700 (0.978–7.430)	
Histology				
Papillary, tubular	1 (reference)	0.119	1 (reference)	0.097
Poorly, mucinous, signet	4.913 (0.664–36.338)		5.715 (0.712–45.861)	
Adjuvant chemotherapy				
Oxaliplatin with 5-FU	1 (reference)	0.621	1 (reference)	0.363
5-FU	0.820 (0.374–1.798)		0.688 (0.307–1.541)	
Conventional Immunoscore				
Low	1 (reference)	0.124		
High	0.504 (0.210–1.207)			
IS-Immunoscore				
Low	1 (reference)	0.004	1 (reference)	0.003
High	0.304 (0.134–0.689)		0.277 (0.117–0.652)	
MMP14^+^ CAF/CAF ratio				
Low	1 (reference)	0.112	1 (reference)	0.022
High	1.936 (0.855–4.384)		2.926 (1.167–7.334)	

A previous preclinical study suggested that MMP14 is associated with resistance to 5-FU treatment in colorectal cancer (32). We therefore analyzed RFS in patients with a high MMP14^+^ CAF/CAF ratio according to adjuvant chemotherapy regimen in order to investigate whether oxaliplatin is able to overcome the poor clinical outcome associated with MMP14^+^ CAFs. There was no significant difference in RFS between patients treated with 5-FU alone and those treated with both oxaliplatin and 5-FU (HR of 0.946, with a 95% CI of 0.355–2.524), however (**Supplementary Figure S5**), indicating that the addition of oxaliplatin to 5-FU did not improve RFS in these patients.

## Discussion

Numerous studies have revealed an important role for nontumor cells in disease progression of solid cancers, but how these cells contribute to tumor recurrence has remained unclear for colorectal cancer. Our study now suggests that CAFs are a key player in postoperative recurrence of stage III colorectal cancer as a result of their expression of MMP14. In addition to our bioinformatics analysis of publicly available transcriptomic data suggesting the clinical relevance of MMP14 in association with tumorigenic cell lineages such as CAFs and TAMs to tumor recurrence, our single cell–based spatial profiling of the TME by mIHC analysis with a digital pathology platform allowed us to clarify that MMP14^+^ CAFs are a determinant of poor survival outcome in stage III colorectal cancer. This mIHC-based spatial profiling also suggested that the tumor aggressiveness conferred by MMP14^+^ CAFs might by explained, at least in part, by promotion of the infiltration of M2-TAMs into the TN by these cells. Of note, our simultaneous evaluation of the Immunoscore revealed that the prognostic role of MMP14^+^ CAFs was independent of that of TILs. We therefore propose a new and improved stratification of stage III colorectal cancer based on both MMP14^+^ CAFs and the Immunoscore.

We found that poor survival was related to MMP14 expression only in CAFs among the various cell types that express this protein. This observation is consistent with previous preclinical findings that expression and activation of MMP14 were detected predominantly in stromal cells (54), with MMP14 expressed in tumor cells remaining largely inactive (54), and that MMP14^+^ CAFs facilitate tumor progression (12, 37). Specific CAF phenotypes have been implicated in tumor progression (7, 55–58). CAFs with a myofibroblastic phenotype (myoCAFs) characterized by a contractile morphology and high αSMA expression were thus found to possess the highest protumorigenic activity, which was driven by endogenous transforming growth factor–β (TGF-β) expression (55). Given that CAFs were defined as αSMA-positive cells in our mIHC analysis, the association of MMP14^+^ CAFs with colorectal cancer progression might be explained by production of the active form of TGF-β mediated by the proteolytic activity of MMP14. MMP14 has also been shown to function as a key collagenase enzyme (59, 60), with ablation of MMP14 in adult mouse fibroblasts giving rise to a marked skin phenotype characterized by increased dermal thickness and tissue stiffness (59). Loss of MMP14 in fibroblasts was shown to affect melanoma growth by altering the composition of the peritumoral extracellular matrix (37, 61). Although we did not examine matrix components such as collagen and laminin, changes in the peritumoral matrix composition may have contributed to the modulation of tumor progression by MMP14. A preclinical study with a breast carcinoma cell line showed that TAMs also express MMP14 (62), consistent with our findings. Of interest, however, MMP14-expressing TAMs were not associated with tumor recurrence in our stage III colorectal cancer cohort. These observations collectively suggest that it is important to focus on specific cell types with regard to the relevance of MMP14 expression to tumor progression. The application of 12-color mIHC as in the present study may thus provide important information regarding the nature of colorectal cancer that complements transcriptome-based findings such as those underlying the CMS categorization.

Evidence has suggested a relation between CAFs and M2-TAMs (63). We here showed that MMP14 expression in CAFs was associated with the infiltration of M2-TAMs into the TN, suggesting that such expression may be a determinant of intratumoral M2-TAM activity in stage III colorectal cancer. The mechanism by which MMP14^+^ CAFs might contribute to M2-TAM accumulation remains unclear, but several previous studies support our current findings. Indeed, MMP14 was shown to increase the amount of the active form of TGF-β through cleavage of the latent form of this growth factor (64, 65), and the active form of TGF-β was found to promote the polarization of TAMs from the antitumor M1 phenotype to the protumorigenic M2 phenotype (66). In addition, a preclinical study showed that inhibition of MMP14 activity resulted in suppression of M2-TAMs and tumor regression (67). Of interest, we found that only M2-TAM infiltration into the TN, not that into the ISA, tended to be associated with a poor prognosis in stage III colorectal cancer, which is consistent with the previous finding that direct physical contact of M2-TAMs with tumor cells was important for the immunosuppressive and protumorigenic activity of M2-TAMs in human gastric cancer (68). In the current study, we adopted CD163 as an M2-TAM marker, although other molecules including CD206 (MRC1) and MRC2 are also expressed on these cells (69, 70) and are indicative of different M2-TAM phenotypes (47, 68, 71). Further research is therefore needed to determine how MMP14 contributes to colorectal cancer progression through regulation of M2-TAM activity.

Our spatial profiling analysis also indicated that T cells in stromal areas, but not those in the TN, play an important role in prevention of tumor recurrence in stage III colorectal cancer, leading us to propose the definition of a modified Immunoscore, the IS-Immunoscore. The reason why T cells in the TN were not associated with survival outcome remains unclear. A reduced tendency of T cells to distribute to the TN is one possible explanation, although some specimens did show substantial infiltration of T cells in this region. The potential mechanisms by which intratumoral T cells might be inactivated in the TN therefore warrant further investigation.

There are several limitations to our study. First, it was retrospective in design and the original cohort was from a single university hospital, with our results thus requiring external validation. However, the results of our independent analysis of transcriptomic data in the TCGA database were consistent with our mIHC findings. Second, we were not able to evaluate CMS categories or genomic alterations such as microsatellite instability and *KRAS* and *BRAF* mutations that are important for precise characterization of colorectal cancer. Further investigations of the role of MMP14^+^ CAFs in the TME should thus include simultaneous transcriptomic and genomic profiling of colorectal tumors. Third, the TME consists of a complex meshwork of extracellular matrix macromolecules and a variety of interspersed cell types (72) including CAFs, blood vessel–associated smooth muscle cells, pericytes, endothelial cells, mesenchymal stem cells, and various kinds of immune cell (73), all of which potentially interact with each other. However, not all immune cell subsets that have been implicated in the survival outcome of colorectal cancer, such as regulatory T cells, were evaluated in our study because of the limited number of markers that could be examined by mIHC (74, 75). Fourth, MMP14 exists as active and inactive forms, with only the former being thought to contribute to the control of tumor growth and cancer cell invasion (76–81). However, immunocytochemistry does not discriminate between these active and inactive forms of MMP14, and our mIHC analysis thus did not allow tracing of cellular MMP14 activity (82). And fifth, several proteins have been identified as markers for CAFs, including αSMA, fibroblast-specific protein 1 (FSP1), vimentin, desmin, fibroblast activation protein (FAP), and platelet-derived growth factor receptor (PDGFR) (22, 83). Although CAFs in CRC were shown to express αSMA at a higher level compared with other fibroblasts (84), we examined only αSMA in our study. Other CAF subsets should thus be examined in the future.

In summary, our study has suggested that MMP14^+^ CAFs play an important role in tumor progression and are therefore a potential therapeutic target in stage III colorectal cancer. Our single cell–based spatial profiling analysis by 12-color mIHC and a digital pathology platform allowed us to identify MMP14^+^ CAFs among the various cellular components of the TME including T cells and TAMs as a determinant of tumorigenic activity. Further studies are warranted to develop new treatment strategies related to the role of MMP14^+^ CAFs in the progression of colorectal cancer.

## Data availability statement

The original contributions presented in the study are included in the article/**Supplementary Material**. Further inquiries can be directed to the corresponding authors.

## Ethics statement

This study was approved by the Institutional Review Board (R02-070) and the Ethics Committee of Kindai University Faculty of Medicine. Written informed consent for participation was not required for this study in accordance with the national legislation and the institutional requirements.

## Author contributions

Conception and design, YM, KH, HK, and KN. Development of methodology, YM, KH, KY, and TT. Acquisition of data (such as management of patients and provision of facilities), YM, KH, HK, and JK. Analysis and interpretation of data (such as statistical analysis, biostatistics, and computational analysis), YM, KH, HK, AI, YC, and KN. Writing, review, and/or revision of the manuscript, YM, KH, and HK. Administrative, technical, or material support (such as reporting or organization of data and database construction), YM, KH, HK, KY, and TT. Study supervision, KH, HK, AI, JK, and KN. Funding, JK, and KN. All authors contributed to the article and approved the submitted version.

## Acknowledgments

We thank Kyoko Itoh of the Department of Pathology and Applied Neurobiology, Kyoto Prefectural University of Medicine, as well as Hiroshi Ogi of SCREEN Holdings Co. Ltd. for technical support.

## Conflict of interest

HK has received consulting fees from Bristol-Myers Squibb Co. Ltd., Eli Lilly Japan K.K., MSD K.K., Ono Pharmaceutical Co. Ltd., Daiichi-Sankyo Co. Ltd., and Taiho Pharmaceutical Co. Ltd.YC honoraria from Bristol-Myers Squibb Co. Ltd., Bayer Yakuhin Ltd., Eli Lilly Japan K.K., MSD K.K., Ono Pharmaceutical Co. Ltd., Chugai Pharmaceutical Co. Ltd., Daiichi Sankyo Co. Ltd., Merck Biopharma Co. Ltd., Takeda Pharmaceutical Co. Ltd., Yakult Pharmaceutical Industry, Teijin Pharma Ltd., and Taiho Pharmaceutical Co. Ltd.YC lecture fees from Glaxo Smith Kline K.K. and Otsuka Pharmaceutical Co. Ltd.YC and research funding from Chugai Pharmaceutical Co. Ltd., Taiho Pharmaceutical Co. Ltd., Kobayashi Pharmaceutical Co. Ltd., and Eisai Co. Ltd. TT has received speaker fees from MSD K.K., Ono Pharmaceutical Co. Ltd., and Bristol-Myers Squibb Co. Ltd. YC has received honoraria from Chugai Pharmaceutical Co. Ltd. KH has received lecture fees from AS ONE Corp., AstraZeneca K.K., Bristol-Myers Squibb Co. Ltd., MSD K.K., and Ono Pharmaceutical Co. Ltd. as well as research funding from AstraZeneca K.K. and MSD K.K. KN has received honoraria from Astellas Pharma Inc., Takeda Pharmaceutical Co. Ltd., Nanzando Co. Ltd., AstraZeneca K.K., Chugai Pharmaceutical Co. Ltd., Roche Diagnostics K.K., MSD K.K., Eli Lilly Japan K.K., Nippon Kayaku Co. Ltd., Daiichi Sankyo Co. Ltd., Novartis Pharma K.K., Kyowa Kirin Co. Ltd., Taiho Pharmaceutical Co. Ltd., Pfizer Japan Inc., AbbVie Inc., Bristol-Myers Squibb Co. Ltd., CareNet Inc., Amgen Inc., Medical Review Co. Ltd., Yodosha Co. Ltd., 3H Clinical Trial Inc., Thermo Fisher Scientific K.K., Hisamitsu Pharmaceutical Co. Inc., Nichi-Iko Pharmaceutical Co. Ltd., Kyorin Pharmaceutical Co. Ltd., Medicus Shuppan Publishers Co. Ltd., Nippon Boehringer Ingelheim Co. Ltd., Nikkei Business Publications Inc., Yomiuri Telecasting Corp., and Medical Mobile Communications Co. Ltd.YC research funding from MSD K.K., AstraZeneca K.K., Pfizer Japan Inc., ICON Japan K.K., Astellas Pharma Inc., Bayer Yakuhin Ltd., Takeda Pharmaceutical Co. Ltd., Novartis Pharma K.K., Otsuka Pharmaceutical Co. Ltd., Eli Lilly Japan K.K., EPS International Co. Ltd., Bristol Myers Squibb Co. Ltd., CMIC Shift Zero K.K., PRA Health Sciences, Taiho Pharmaceutical Co. Ltd., Eisai Co. Ltd., Merck Biopharma Co. Ltd., Parexel International Corp., Mochida Pharmaceutical Co. Ltd., Covance Japan Inc., Ono Pharmaceutical Co. Ltd., Kissei Pharmaceutical Co. Ltd., Medical Research Support, Sysmex Corp., GlaxoSmithKline K.K., Sanofi K.K., A2 Healthcare Corp., Kyowa Hakko Kirin Co. Ltd., Syneos Health, AbbVie Inc., EPS Corp., Pfizer R&D Japan G.K., Chugai Pharmaceutical Co. Ltd., Daiichi Sankyo Co. Ltd., PPD-SNBL K.K., Nippon Boehringer Ingelheim Co. Ltd., IQVIA Services Japan K.K./Quintiles Inc., Japan Clinical Research Operations, and SymBio Pharmaceuticals Ltd.YC and consulting fees from Astellas Pharma Inc., Takeda Pharmaceutical Co. Ltd., Eli Lilly Japan K.K., Pfizer Japan Inc., Kyorin Pharmaceutical Co. Ltd., and Ono Pharmaceutical Co. Ltd.

The remaining authors declare that the research was conducted in the absence of any commercial or financial relationships that could be construed as a potential conflict of interest.

## Publisher’s note

All claims expressed in this article are solely those of the authors and do not necessarily represent those of their affiliated organizations, or those of the publisher, the editors and the reviewers. Any product that may be evaluated in this article, or claim that may be made by its manufacturer, is not guaranteed or endorsed by the publisher.
