# Supplementary material for: Contribution of MMP14-expressing cancer-associated fibroblasts in the tumor immune microenvironment to progression of colorectal cancer
Source: Front Oncol. 2022 Aug 16;12:956270. doi: 10.3389/fonc.2022.956270 (PMC9424903; doi:10.3389/fonc.2022.956270)
Supplement: Supplementary file 1 [file DataSheet_1.pdf]

Supplementary Table S1. Details of the mIHC panel adopted in this study.

	Round 1	Round 2	Round 3	Round 4	Round 5	Round 6	Round 7
Primary Ab	[Hematoxylin]	CD80	CD3	CD31	CD45	CD33	CD8
Supplier	Dako	R&D Systems	Thermo Scientific	Dako	Thermo Scientific	Abcam	Dako
Clone/catalog#	S3301	MAB140-37711	SP7	JC70A	H130	SP266	C8/144B
Dilution		1:30	1:150	Undiluted	1:100	1:50	1:100
Reaction	1 min	RT, 30 min	RT, 30 min	RT, 30 min	RT, 30 min	RT, 30 min	RT, 30 min
Histofine		Anti-mouse	Anti-rabbit	Anti-mouse	Anti-mouse	Anti-rabbit	Anti-mouse
Reaction		RT, 30 min	RT, 30 min	RT, 30 min	RT, 30 min	RT, 30 min	RT, 30 min
AEC reaction time		20 min	20 min	20 min	40 min	20 min	20 min

	Round 8	Round 9	Round 10	Round 11	Round 12	Round 13
Primary Ab	CD68	MMP14	HLA-DR	αSMA	CD163	PanCK
Supplier	Abcam	Abcam	Novus Biological	Abcam	Thermo Scientific	Leica Biosystems
Clone/catalog#	PG-M1	EP1264Y	SPM288	ab5694	10D6	AE1/AE3
Dilution	1:50	1:250	1:400	1:400	1:100	1:200
Reaction	RT, 30 min	RT, 30 min	RT, 30 min	RT, 30 min	RT, 30 min	RT, 30 min
Histofine	Anti-mouse	Anti-rabbit	Anti-mouse	Anti-rabbit	Anti-mouse	Anti-mouse
Reaction	RT, 30 min	RT, 30 min	RT, 30 min	RT, 30 min	RT, 30 min	RT, 30 min
AEC reaction time	20 min	20 min	10 min	20 min	20 min	10 min

Abbreviations not defined in text: Ab, antibody; AEC, alcohol-soluble peroxidase substrate 3-aminomethyl carbazolezole; RT, room temperature.

Supplementary Table S2. Correlation between immune cell density in the TN and the MMP14$^+$ CAF/CAF ratio.

Immune cell subset	Spearman’s correlation coefficient	*P* value
CD3$^+$ T cell	0.111	0.309
CD8$^+$ T cell	0.089	0.417
M1-TAM	0.210	0.052
M2-TAM	0.334	0.002
MDSC	0.162	0.136

Supplementary Table S3. Correlation between immune cell density in the ISA and the MMP14$^+$ CAF/CAF ratio.

Immune cell subset	Spearman's correlation coefficient	*P* value
CD3$^+$ T cell	0.149	0.173
CD8$^+$ T cell	0.038	0.732
M1-TAM	0.093	0.394
M2-TAM	0.394	<0.001
MDSC	0.103	0.346

Supplementary Figure S1. Flow diagram for patient selection.

Supplementary Figure S2. Representative images of mIHC analysis. **(A)** Hematoxylin and original alcohol-soluble peroxidase substrate 3-aminomethyl carbazolezole (AEC) staining images for mIHC analysis. Scale bar, 400 μm. **(B)** Representative hematoxylin images for the Tissue Segmentation method. Regions of interest were randomly selected for the invasive front (IF) and the center of the tumor (CT). The Tissue Segmentation method (17) was adopted to extract the intratumoral stromal area (ISA) and the tumor cell nest (TN) from CT. The same method was applied to extract IF as the area within 300 μm external to the boundary of the tumor cell area with the surrounding connective tissue. **(C)** Cell distribution according to areas determined by Tissue Segmentation. The distribution of tumor cells ($CD31^{-}CD45^{-}PanCK^{+}\alpha SMA^{-}$) and CAFs ($CD31^{-}CD45^{-}PanCK^{-}\alpha SMA^{+}$) was evaluated in the TN and ISA by FCS Express 7 Image Cytometry.

Supplementary Figure S3. Kaplan-Meier curves for RFS according to cell subsets. **(A)** Kaplan-Meier curves for RFS based on median values of the $MMP14^{+}$ TAM/TAM ratio (upper panels) or the $MMP14^{+}$ $CD3^{+}$ T cell/$CD3^{+}$ T cell ratio (lower panels) in the ISA (left panels) or the TN (right panels). **(B)** Kaplan-Meier curves for RFS based on the median value of overall CAF density. **(C and D)** Correlation between CAF density and M2-TAM density in the ISA (C) or TN (D). Each dot represents one patient. Regression lines are also shown in red.

Supplementary Figure S4. Relation between T cell distribution in tumor tissue and RFS. **(A)** Kaplan-Meier curves for RFS based on the conventional Immunoscore. **(B and C)** Kaplan-Meier curves for RFS based on the median values of $CD3^{+}$ T cell (B) or $CD8^{+}$ T cell (C) density in the IF (left), ISA (middle), or TN (right) regions. **(D)** Box

plots for the proportion of $CD3^+$ T cells among $CD45^+$ cells in the IF, ISA, and TN regions. The boxes represent the median and upper and lower quartiles, and the whiskers represent the range. The *P* values were determined with the Steel-Dwass test. **(E)** Kaplan-Meier curves for RFS according to the IS-Immunoscore.

Supplementary Figure S5. Kaplan-Meier curves for RFS based on adjuvant chemotherapy regimens among patients with a high $MMP14^+$ CAF/CAF ratio relative to the median value. OX, oxaliplatin.
