# Supplementary figures and images for: Contribution of MMP14-expressing cancer-associated fibroblasts in the tumor immune microenvironment to progression of colorectal cancer
Source: Front Oncol. 2022 Aug 16;12:956270. doi: 10.3389/fonc.2022.956270 (PMC9424903; doi:10.3389/fonc.2022.956270)

Supplementary Figure 1

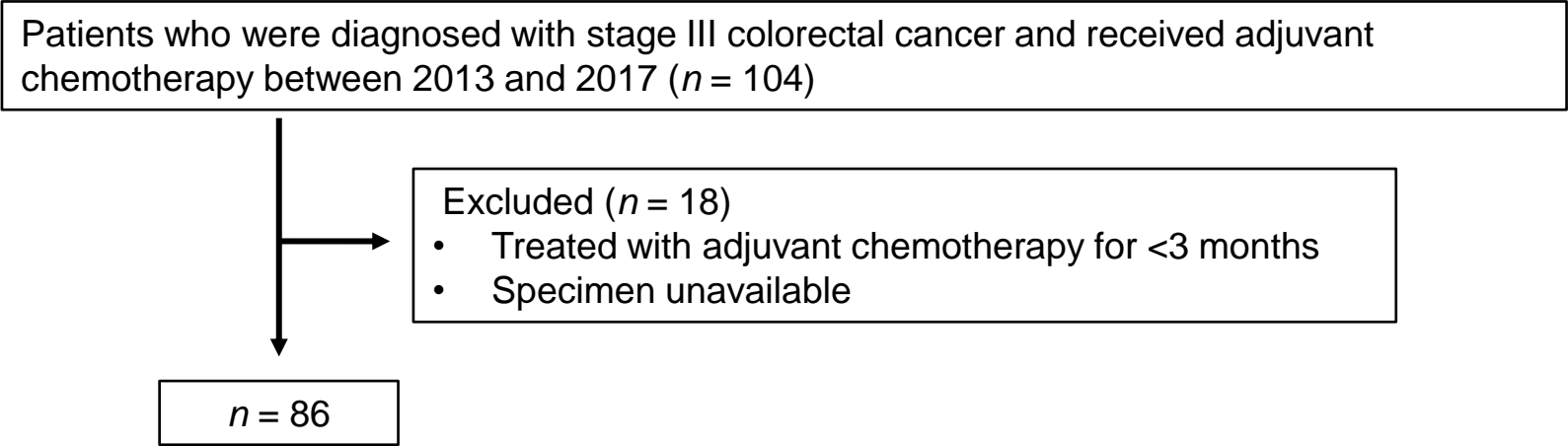

Supplement: Supplementary file 2 [file Image_1.pdf]

Supplementary Figure 2

A

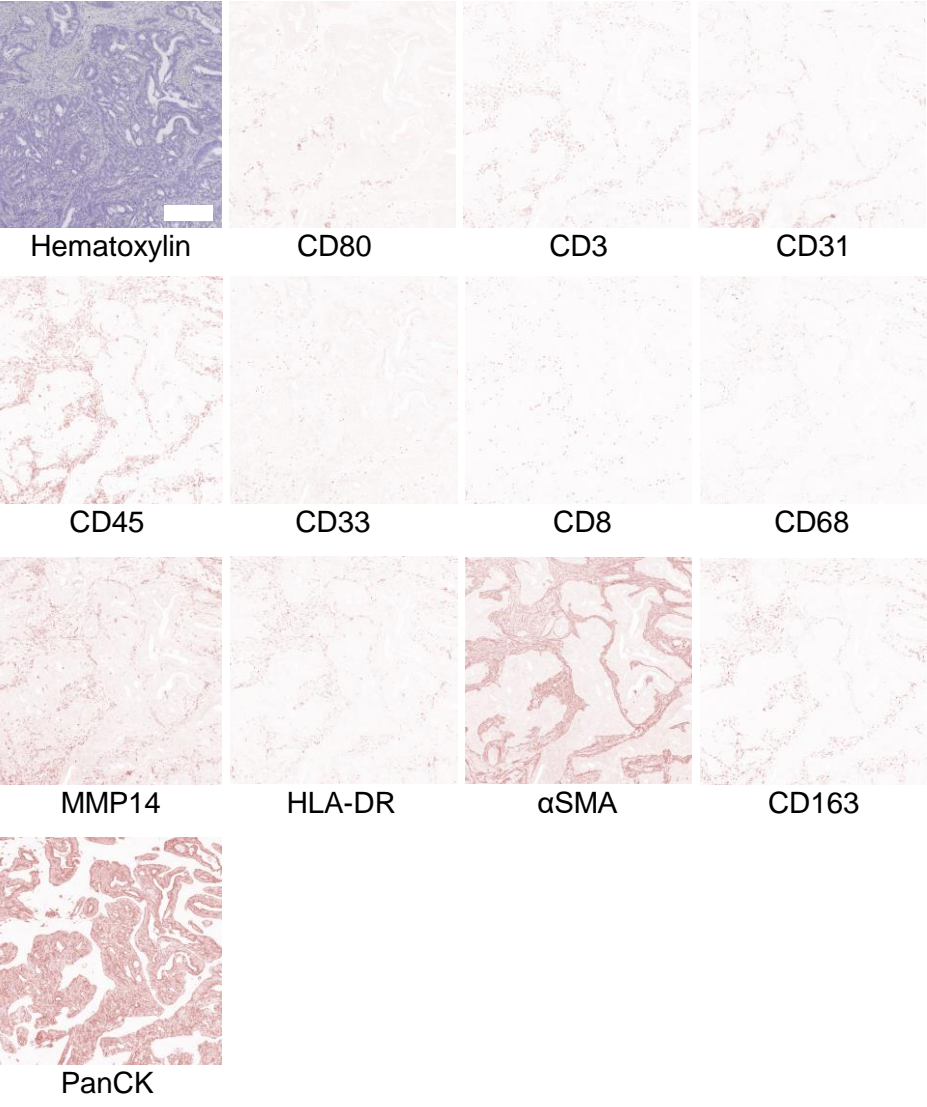

B

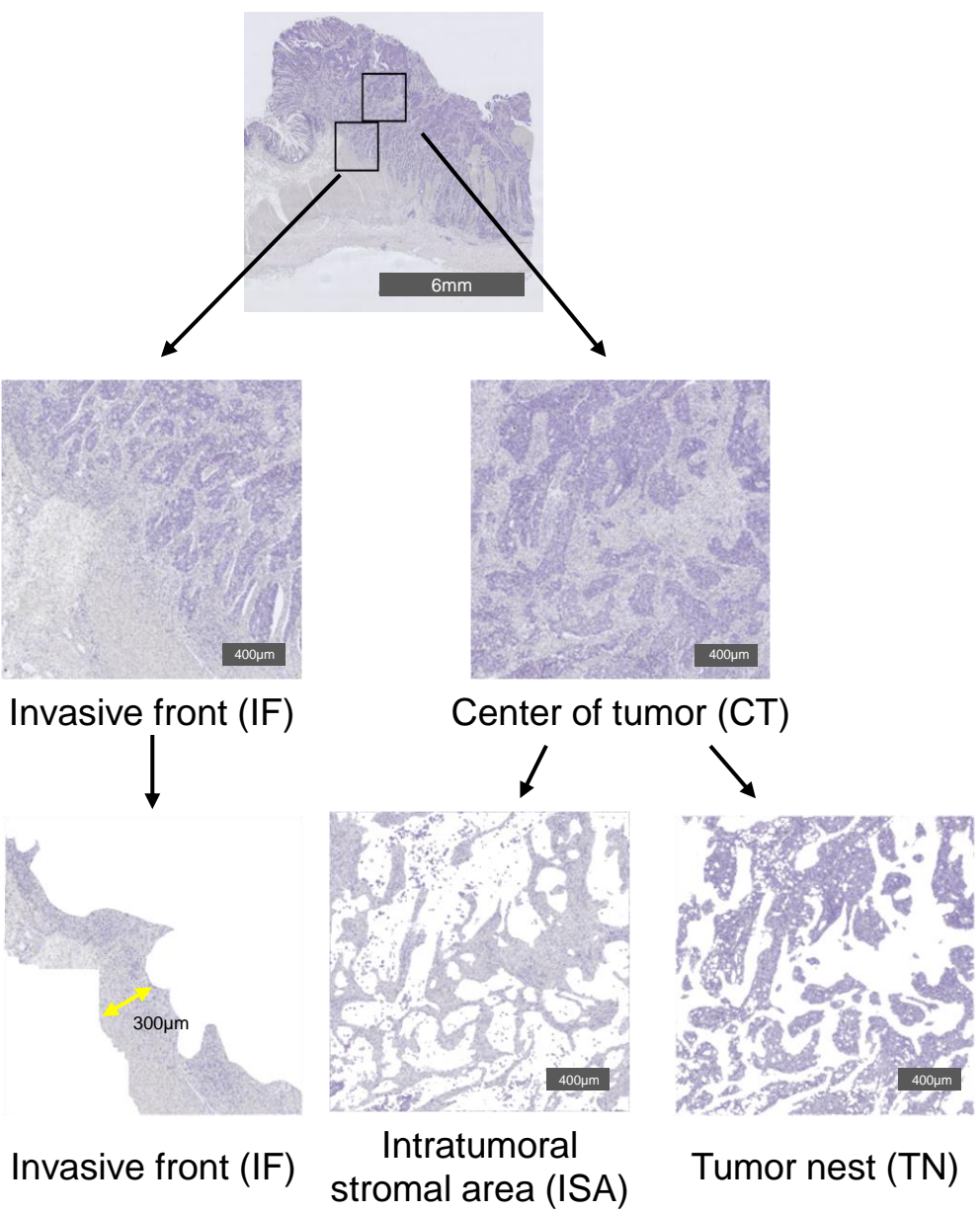

C

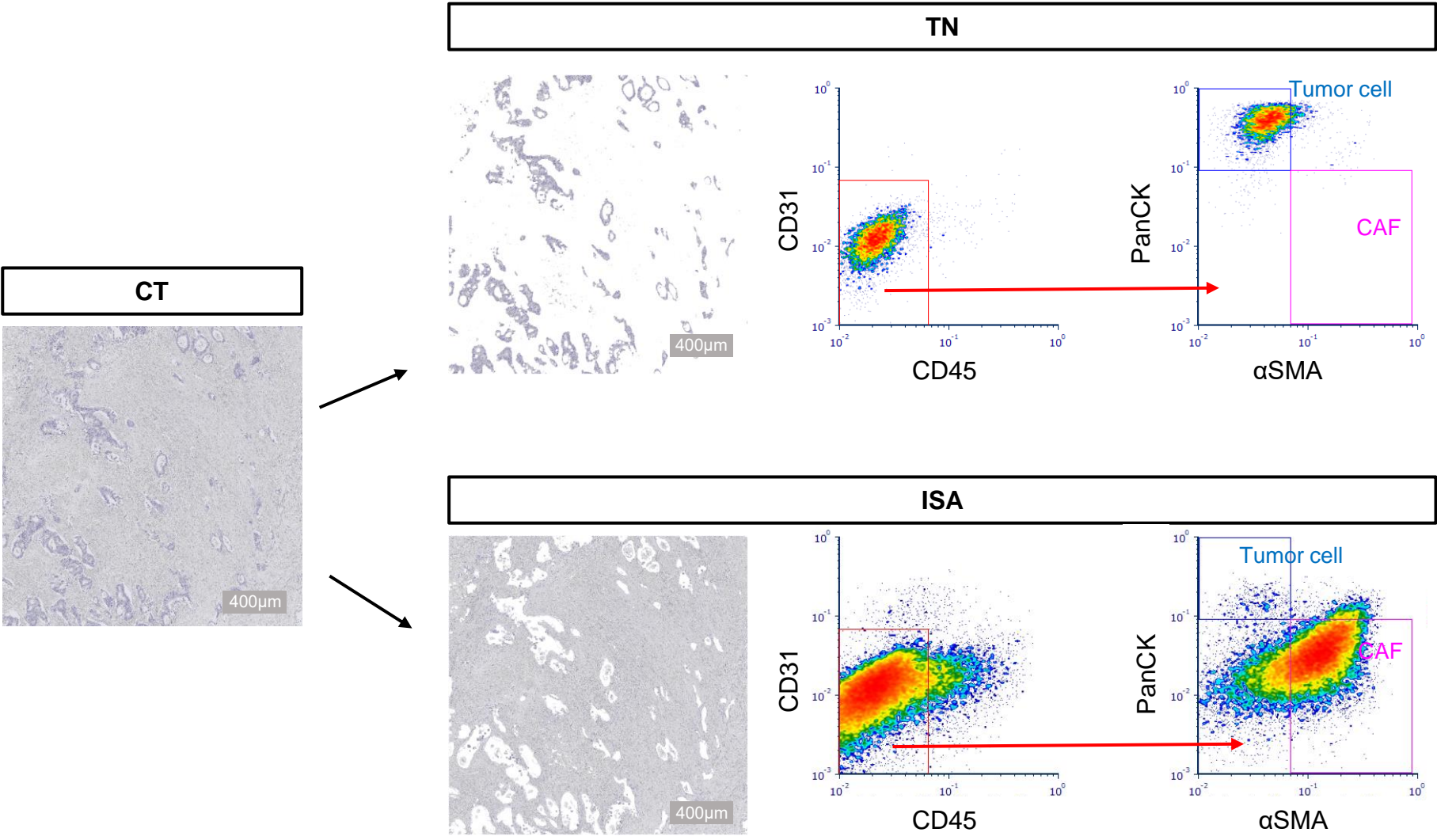

Supplement: Supplementary file 3 [file Image_2.pdf]

Supplementary Figure 3

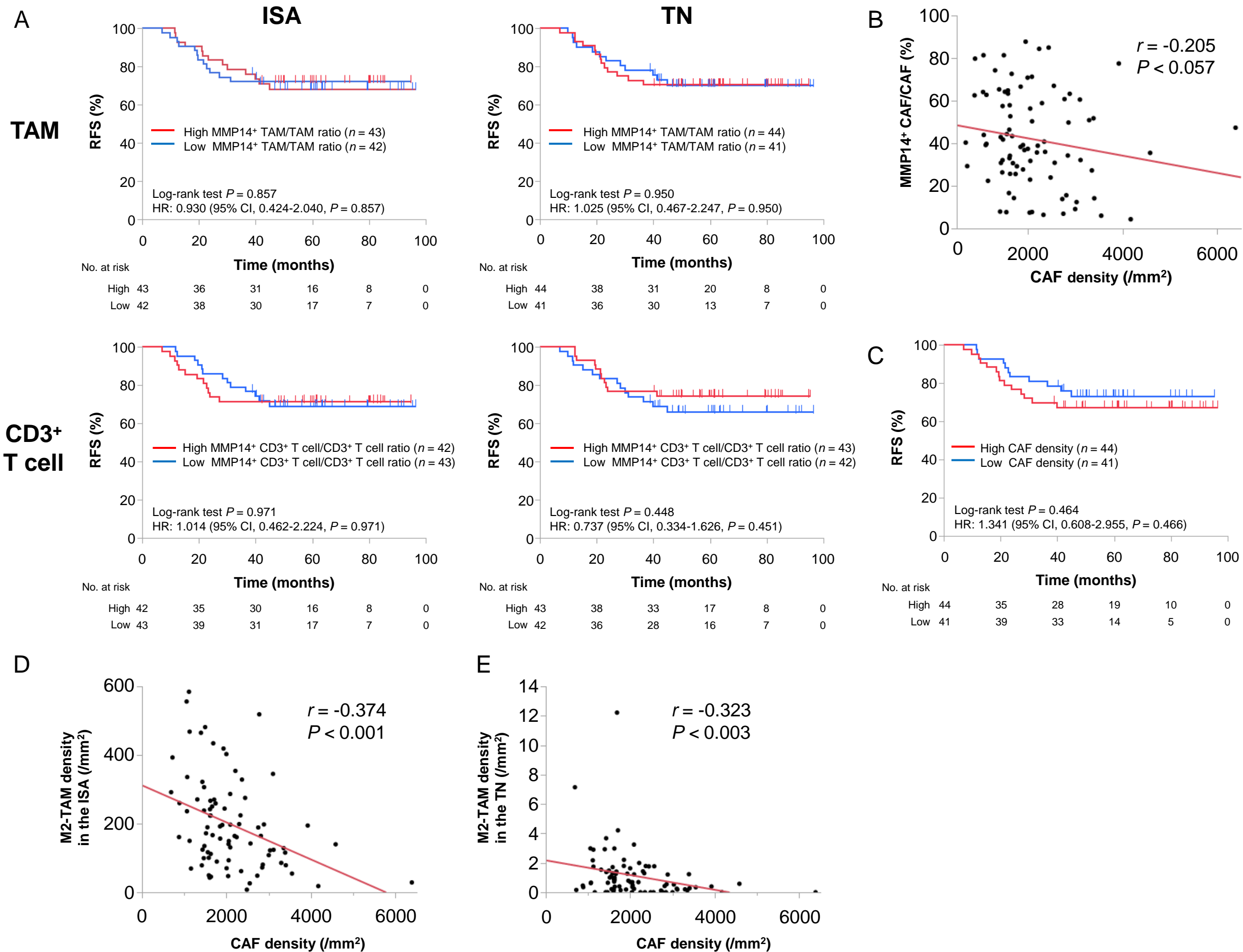

Supplement: Supplementary file 4 [file Image_3.pdf]

Supplementary Figure 4

A Conventional Immunoscore

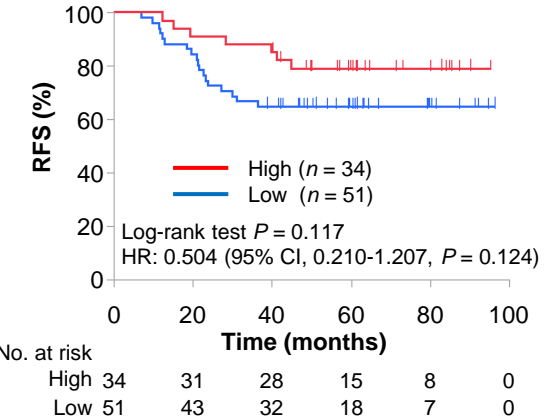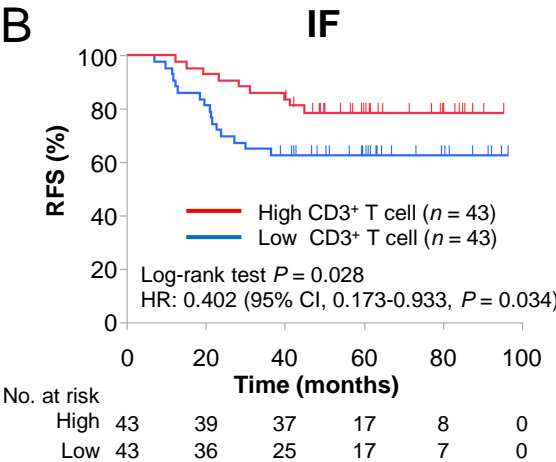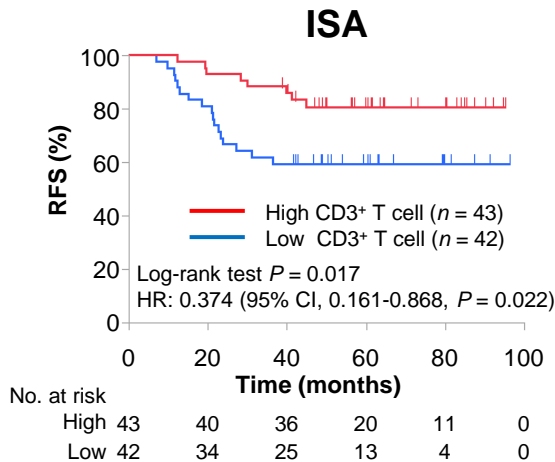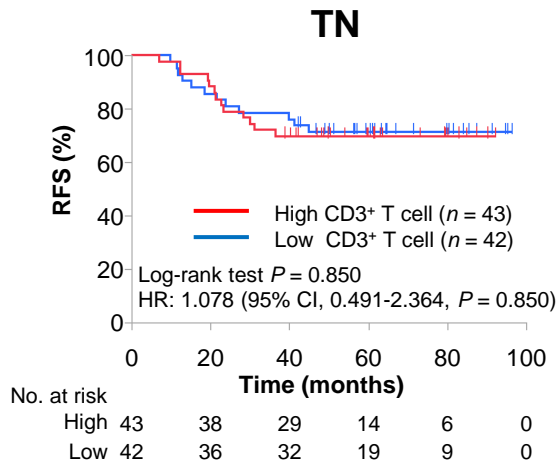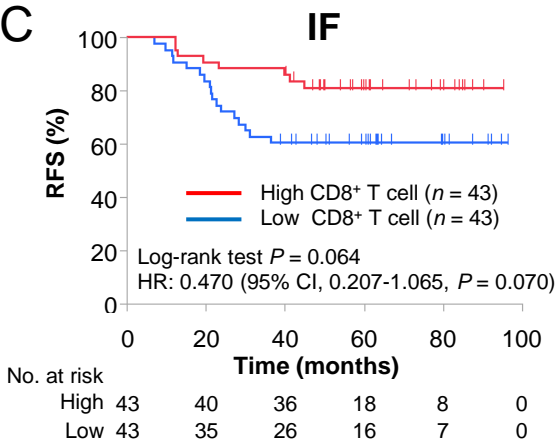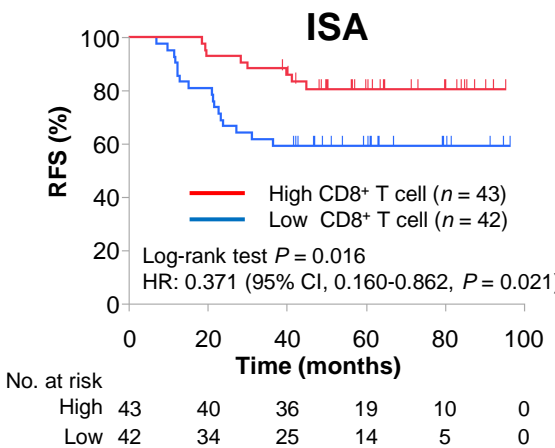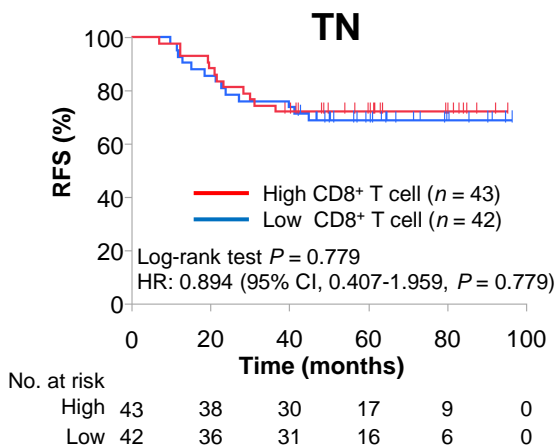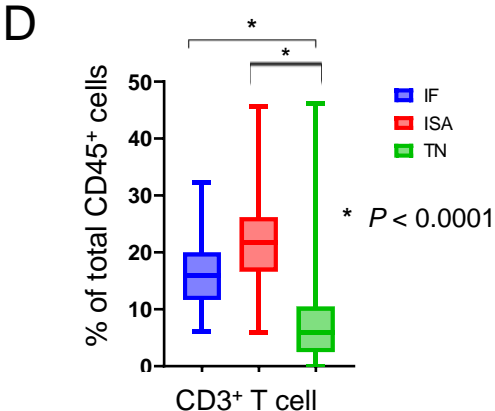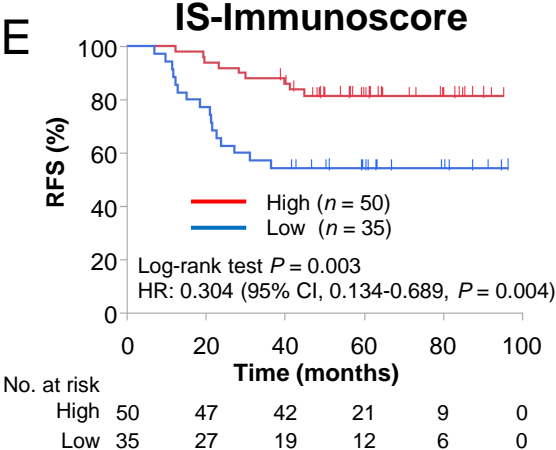

Supplement: Supplementary file 5 [file Image_4.pdf]

Supplementary Figure 5

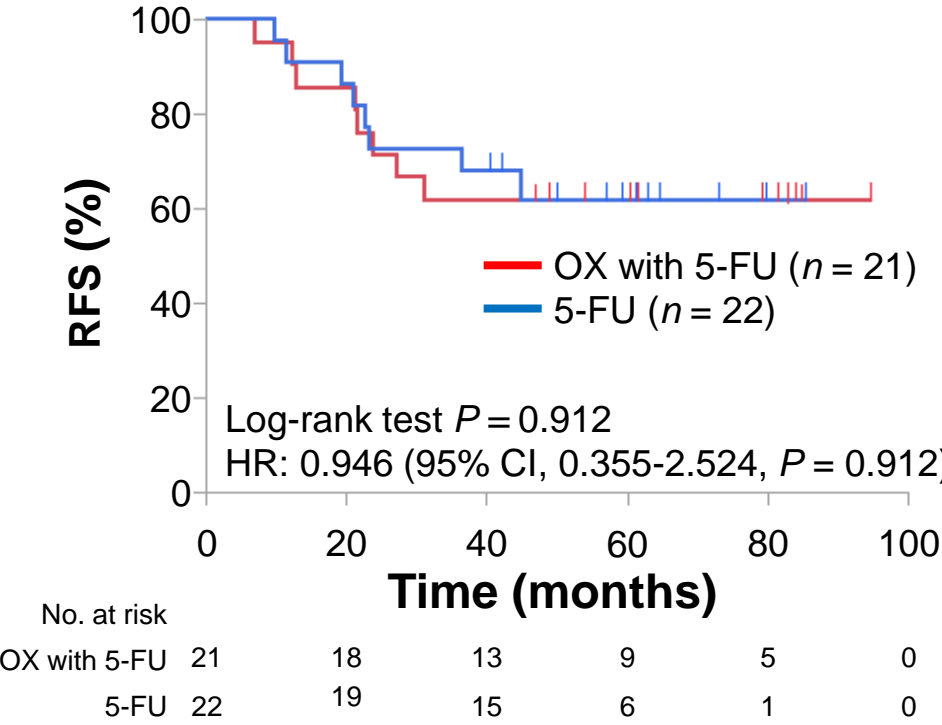

Supplement: Supplementary file 6 [file Image_5.pdf]
